# Digital Health Applications to Establish a Remote Diagnosis of Orthopedic Knee Disorders: Scoping Review

**DOI:** 10.2196/40504

**Published:** 2023-02-09

**Authors:** Sander C van Eijck, Daan M Janssen, Maria C van der Steen, Eugenie J L G Delvaux, Johannes G E Hendriks, Rob P A Janssen

**Affiliations:** 1 Department of Orthopedic Surgery & Trauma Máxima Medical Center Veldhoven Netherlands; 2 Department of Orthopedic Surgery & Trauma Catharina Hospital Eindhoven Netherlands; 3 Medical Library Máxima Medical Center Veldhoven Netherlands; 4 Orthopedic Biomechanics, Department of Biomedical Engineering Eindhoven University of Technology Eindhoven Netherlands; 5 Value-Based Health Care, Department of Paramedical Sciences Fontys University of Applied Sciences Eindhoven Netherlands

**Keywords:** orthopedic surgery, eHealth, digital health, mobile health, mHealth, telemedicine, artificial intelligence, diagnosis, remote patient management, musculoskeletal system, knee, mobile phone

## Abstract

**Background:**

Knee pain is highly prevalent worldwide, and this number is expected to rise in the future. The COVID-19 outbreak, in combination with the aging population, rising health care costs, and the need to make health care more accessible worldwide, has led to an increasing demand for digital health care applications to deliver care for patients with musculoskeletal conditions. Digital health and other forms of telemedicine can add value in optimizing health care for patients and health care providers. This might reduce health care costs and make health care more accessible while maintaining a high level of quality. Although expectations are high, there is currently no overview comparing digital health applications with face-to-face contact in clinical trials to establish a primary knee diagnosis in orthopedic surgery.

**Objective:**

This study aimed to investigate the currently available digital health and telemedicine applications to establish a primary knee diagnosis in orthopedic surgery in the general population in comparison with imaging or face-to-face contact between patients and physicians.

**Methods:**

A scoping review was conducted using the PubMed and Embase databases according to the PRISMA-ScR (Preferred Reporting Items for Systematic Reviews and Meta-Analyses extension for Scoping Reviews) statement. The inclusion criteria were studies reporting methods to determine a primary knee diagnosis in orthopedic surgery using digital health or telemedicine. On April 28 and 29, 2021, searches were conducted in PubMed (MEDLINE) and Embase. Data charting was conducted using a predefined form and included details on general study information, study population, type of application, comparator, analyses, and key findings. A risk-of-bias analysis was not deemed relevant considering the scoping review design of the study.

**Results:**

After screening 5639 articles, 7 (0.12%) were included. In total, 2 categories to determine a primary diagnosis were identified: screening studies (4/7, 57%) and decision support studies (3/7, 43%). There was great heterogeneity in the included studies in algorithms used, disorders, input parameters, and outcome measurements. No more than 25 knee disorders were included in the studies. The included studies showed a relatively high sensitivity (67%-91%). The accuracy of the different studies was generally lower, with a specificity of 27% to 48% for decision support studies and 73% to 96% for screening studies.

**Conclusions:**

This scoping review shows that there are a limited number of available applications to establish a remote diagnosis of knee disorders in orthopedic surgery. To date, there is limited evidence that digital health applications can assist patients or orthopedic surgeons in establishing the primary diagnosis of knee disorders. Future research should aim to integrate multiple sources of information and a standardized study design with close collaboration among clinicians, data scientists, data managers, lawyers, and service users to create reliable and secure databases.

## Introduction

### Background

The World Health Organization defines digital health as “a broad umbrella term encompassing eHealth (which includes mobile Health (mHealth)), as well as emerging areas, such as the use of advanced computing sciences in ‘big data’, genomics and artificial intelligence” [1]. These include—but are not limited to—web applications, health IT, wearable devices, personalized medicine, telehealth, telemedicine, gait analysis, and artificial intelligence (AI) techniques such as machine learning and deep learning [2,3]. Over the last years, there has been an increase in the use of digital health applications in the field of orthopedics for gait analysis, diagnosis, imaging, computer-assisted surgery, and telerehabilitation [4-8]. Digital health has been shown to reduce the number of patient visits and present a valuable tool for the continuity of health care without decreasing patient or health care provider satisfaction [9,10].

The years 2020 and 2021 were characterized by the outbreak of COVID-19, with the need to minimize patient contact to comply with social distancing measures. Owing to this pandemic, the urge rose for hospitals to minimize face-to-face contact between patients and health care providers [11]. This, in combination with the increase in the prevalence of musculoskeletal diseases, rising health care costs, and the need for more accessible health care worldwide, increases the demand for digital health solutions [12-14]. Remote patient monitoring facilitated by digital health solutions, such as making a primary diagnosis without face-to-face contact, for medical problems with high prevalence would provide an opportunity for continuation of care and might be able to make care more accessible and affordable worldwide [14].

Knee pain is an example of a high-prevalence musculoskeletal disease, with a lifetime prevalence of >50% in adults in Western countries [15,16]. Owing to an aging population, it is expected that the number of musculoskeletal complaints for the knee will rise in the future [17]. Although the expectations for digital health solutions are high, there is currently no overview available of the literature on the use of digital health applications to assist in establishing a primary diagnosis in orthopedic surgery that compares existing applications with conventional imaging techniques or face-to-face contact.

### Objectives

The aim of this scoping review was to provide an overview of the available literature on digital health applications in comparison with a clinical gold standard such as face-to-face contact or imaging to establish a remote diagnosis of knee disorders in orthopedic surgery in the general population. We wish to provide the reader with an overview of the investigated knee disorders, input parameters used, and underlying methods. Furthermore, accuracy measures—sensitivity and specificity—were used to determine whether it is possible to reliably establish a remote diagnosis of knee disorders that can be used in clinical practice. As such, this scoping review will provide a better understanding of what is currently possible with digital health in clinical practice and which areas need more research to develop adequate digital health applications for undiagnosed patients with knee pain.

## Methods

### Search Methods

This scoping review was conducted in accordance with the PRISMA-ScR (Preferred Reporting Items for Systematic Reviews and Meta-Analyses extension for Scoping Reviews) guidelines (Multimedia Appendices 1 and 2 [18]). An information specialist (EJLGD) performed a systematic literature search in the medical databases PubMed (MEDLINE) and Embase (Ovid) on April 28 and 29, 2021, as shown in Multimedia Appendix 3. The applied terms, including synonyms and closely related words, were “Telemedicine/eHealth, Knee, Knee Joint, Diagnosis.” The complete list of Medical Subject Heading terms can be found in Multimedia Appendix 3. A search of the references of the full-text studies was also performed. There were no limitations regarding the year of publication. The inclusion and exclusion criteria for screening identified articles for eligibility are listed in Textbox 1.

**Inclusion and exclusion criteria.**

**Inclusion criteria**

- Studies reporting methods to establish (in part) a primary diagnosis of knee disorders using digital health for orthopedic surgery
- Application compared with face-to-face physician-patient contact (or conventional validated diagnostic tools such as goniometers and imaging)
- Digital health applications (remote or web-based care in any form including, but not limited to, mobile apps, video, telephone, and internet-based or telemedicine tools that can be used digitally)
- Aim to develop or validate a digital health application and evaluate one or more measurement properties (ie, sensitivity, specificity, area under the curve, or inter- or intraclass correlation coefficient) of a digital health application for diagnosis of knee disorders in orthopedic surgery
- Minimum of 1 study participant
- Studies with humans
- Full-length publication in a peer-reviewed journal
- Languages: English, German, or Dutch
- Patient population: all ages

**Exclusion criteria**

- Rehabilitation and nondiagnostic follow-up studies
- Primary diagnosis solely via imaging, joint puncture, or laboratory tests
- Digital health applications without input from the patient or non–health care provider to establish a primary diagnosis
- Digital sensors (including wearables) as the main focus of the study
- Cadaver and animal studies
- Editorials, conference papers, and published abstract papers

### Article Screening

In total, 2 authors (SCvE and DMJ) independently performed abstract screening for eligibility via the web-based program Rayyan (Qatar Computing Research Institute) [19]. Full texts of all the eligible abstracts were retrieved and reviewed independently by both authors. If there was any discussion about including or excluding a study, a third more senior author was consulted (RPAJ), and it was resolved through discussion. As the aim of this study was to compare applications with face-to-face contact, we decided not to include conference papers and abstracts and only include peer-reviewed published articles to be able to look into the details of the methodology and subsequent results of the different studies.

### Data Charting

The data were independently charted via a predefined form by 2 authors (SCvE and DMJ) and verified by a third author (RJ) using Microsoft Excel (Microsoft Corp). The extracted data included details on general study information, study population, type of application, comparator, analyses, and key findings. See Multimedia Appendix 4 for a full list of the extracted parameters.

### Synthesis of Results

A narrative synthesis was conducted to organize, describe, and interpret the results. Studies were categorized by aim of the application, namely, screening or generating a differential diagnosis. The extracted data were summarized in tables to provide an overview of the examined knee disorders, input parameters used, and algorithms underlying the different applications. Reported statistical outcome measurements such as sensitivity, specificity, and area under the curve (AUC) were used to estimate whether the application was accurate enough for potential use in clinical practice and could be used to establish a primary diagnosis of knee disorders. A risk-of-bias analysis was not deemed relevant considering the scoping review design of the study.

## Results

### Overview

**Figure 1:**
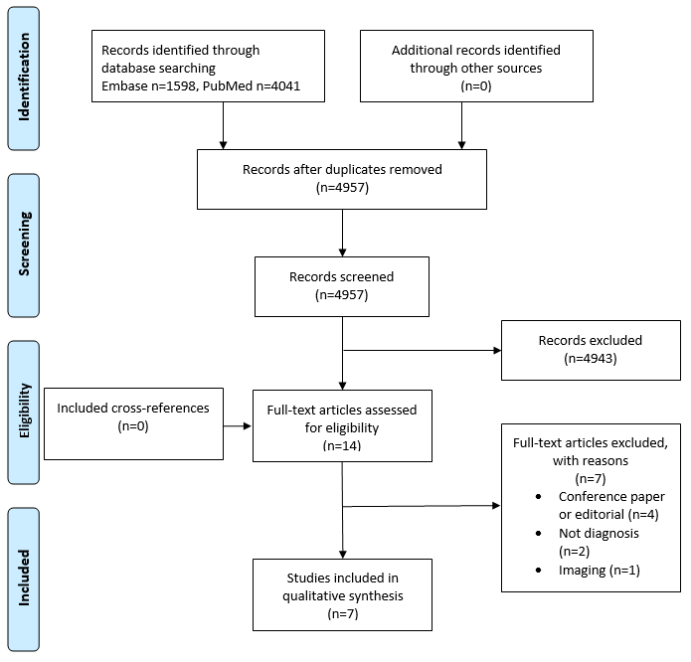
PRISMA (Preferred Reporting Items for Systematic Reviews and Meta-Analyses) flowchart.

Figure 1 shows the results of the PRISMA (Preferred Reporting Items for Systematic Reviews and Meta-Analyses) search strategy. After screening abstracts and full-text articles for inclusion and exclusion, 7 studies were included (Table 1) [20-26]. All the relevant articles yielded by the search were published after 2008.

The articles could be divided into applications that were interested in 2 different phases of the diagnostic process: screening and decision support. The number of patients, methods, and aim of the study are summarized in Table 1. The aim of screening studies was to detect a specific disorder in the general population. Decision support studies generated a differential diagnosis to aid in the process of determining a final diagnosis. Most screening applications (3/4, 75%) focused on the detection of osteoarthritis (OA) [23-25], and 25% (1/4) focused on screening for meniscal tears [26]. The decision support studies, in contrast, focused on multiple knee disorders. The number of diagnoses considered by the separate decision support applications ranged from 12 to 25 (Textbox 2) [20-22].

**Table 1:** Characteristics and aims of the included studies (N=7).

Study, year of publication		Age (years), mean (range)	Female participants, n (%)	Patients, n	Method	Disease	Aim	Comparator
**Decision support studies**								
	Bisson et al [20], 2014	47 (18-81)	255 (48)	615; final analysis: 527	Web-based symptom checker	Multiple knee disorders^a^	Establish a differential diagnosis of knee injuries	Orthopedic surgeon
	Bisson et al [21], 2016	48 (18-76)	165 (50)	790; final analysis: 328	Web-based symptom checker	Multiple knee disorders^a^	Establish a differential diagnosis of knee injuries and let patients determine the right diagnosis from a list	Orthopedic surgeon
	Elkin et al [22], 2018	44 (1-84)	Not available (50)	469	Web interface with 26 questions and 10 possible diagnoses analyzed using AI^b^	Multiple knee disorders^a^	Establish a differential diagnosis list comparing heuristic versus Bayesian algorithms	Diagnosis by 2 orthopedic surgeons based on the same 26-item questionnaire
**Screening studies**								
	Lim et al [23], 2019	Not available	Not available	5749	Deep learning algorithm	Osteoarthritis	Prediction of osteoarthritis in a nationwide database	Medical record or diagnosis of osteoarthritis
	Ratzlaff et al [24], 2012	63.3 (not available)	Not available (54)	100 (200 knees and 200 hips)	Web-based questionnaire	Knee and hip osteoarthritis	Detecting knee and hip osteoarthritis	Experienced orthopedic physiotherapist
	Roux et al [25], 2008	58 (not available)	Not available (68)	Initial screening: 1380; conformation analysis: 109; control group: 140	Telephone questionnaire	Knee and hip osteoarthritis	Detecting knee and hip osteoarthritis in the general population	Rheumatologist
	Snoeker et al [26], 2015	47 (18-84)	50 (48)	121; after final analysis: 117	Questionnaire with 1 physical examination test	Meniscal tears	Detect meniscal tears in a primary care population	MRI^c^

^a^See Textbox 2 for an overview of the included disorders.

^b^AI: artificial intelligence.

^c^MRI: magnetic resonance imaging.

**Diseases included in the decision support studies.**

**Included knee disorders**

- Anterior cruciate ligament tear [20-22]
- Iliotibial band friction syndrome [20,21]
- Lateral collateral ligament tear [20,21]
- Medial collateral ligament tear [20-22]
- Meniscal tear [20-22]
- Osgood-Schlatter disease [20,21]
- Osteoarthritis [20-22]
- Osteoarthritis exacerbation [20-22]
- Osteochondritis dissecans [20,21]
- Patellar arthritis [20-22]
- Patellar arthritis exacerbation [20-22]
- Patellar chondromalacia and patellofemoral syndrome [20-22]
- Patellar contusion and saphenous nerve contusion [20-22]
- Patellar instability [20-22]
- Patellar tendinitis [20-22]
- Patellar tendon rupture (partial or complete) [20,21]
- Plica syndrome [20,21]
- Popliteal cyst [20-22]
- Posterior cruciate ligament tear [20,21]
- Prepatellar bursitis [20,21]
- Quadriceps tendinitis [20,21]
- Quadriceps tendon tear (partial or complete) [20,21]
- Rheumatoid arthritis [20,21]
- Stress fracture [20,21]
- Trochlear chondromalacia [20,21]

### Input and Underlying Algorithms Used for the Applications

Questionnaires were the main source of input for both the screening and decision support applications. The input parameters for the different applications are presented in Textbox 3 [20-26]. Most studies (6/7, 86%) used basic demographic factors as input [20-25]. There was a large variety of questions related to knee injuries among the studies. Only a limited number of studies (3/7, 43%) included lifestyle factors in their applications [23,24,26]. In total, 14% (1/7) of the studies used a physical test as input [26]. The included studies used no other input sources to establish a diagnosis. The algorithms to establish the diagnosis varied from simple skip logic to sophisticated AI techniques such as deep neural networks (DNNs; see the following sections for an elaborate description). A summary of the results of the individual studies is presented in Table 2. This table shows that the sensitivity of most studies was high, with low specificity for most of the decision support studies in contrast. In addition, the decision support studies (3/7, 43%) showed a low specificity in ranking the correct diagnosis on top in comparison with an orthopedic surgeon. To determine whether applications can be used in clinical practice, it is important to provide a more detailed description of the screening and decision support studies.

**Input parameters that were used in the included studies.**

**Demographic factors**

- Age [20-23,26]
- BMI [20-24]
- Chronic diseases [23,24]
- Educational level [23]
- Gender [20-23,26]
- Household income [23]
- Marital status [23]
- Region [23]

**Injury-related factors**

- Deep squat test [26]
- Discoloration [26]
- Duration of pain [22]
- Location of pain [20-22]
- Pain [25]
- Pain in hand or wrist [22]
- Pain during activities or weight bearing [22,25]
- Previous diagnosis [24,25]
- Previous treatments [20-22]
- Swelling or effusion [22,25,26]
- Type of injury [20-22,24]
- Warmth [26]

**Lifestyle factors**

- Alcohol intake [23]
- Occupation [24]
- Physical activity [23]
- Smoking status [23]
- Self-reported health status [23]
- Sports [26]

**Table 2:** Summary of the results of the included studies (N=7).

Study, year of publication		Absolute results	Statistical measurement results
**Decision support studies**			
	Bisson et al [20], 2014	674 out of 758 diagnoses generated by the program contained the diagnosis of the physician 674 correct matches out of a total of 2512 differential diagnoses	Sensitivity: 89% Specificity: 27%
	Bisson et al [21], 2016	496 out of 543 diagnoses generated by the program contained the diagnosis of the physician 496 correct matches out of a total of 2161 differential diagnoses 315 out of 543 times the patient was able to identify the diagnosis from the list 315 out of 653 selected diagnoses by the patient were indeed the physician’s diagnosis	Sensitivity: 91% Specificity: 23% Sensitivity of the tool when used by patients: 58% Specificity of the tool when used by patients: 48%
	Elkin et al [22], 2018	Mean rank of true diagnosis: Model 3: 2.215 Model 4: 2.522	Number of patients who had the true diagnosis at rank 1 in the expert model: Model 1: 203 out of 469 (43.3%) Model 2: 203 out of 469 (43.3%) Model 3: 224 out of 469 (47.8%) Model 4: 191 out of 469 (40.7%)
**Screening studies**			
	Lim et al [23], 2019	270 out of 405 patients with OA^a^ correctly labeled 1137 out of 1550 patients without OA correctly labeled	Sensitivity: 67% Specificity: 73% Area under the curve: 76% Accuracy: 71.97%
	Ratzlaff et al [24], 2012	25 out of 34 patients with clinical knee OA had a positive test 148 out of 166 patients with a negative test did not have knee OA	For knee OA: Sensitivity: 73% Specificity: 96% Positive predictive value: 86% Negative predictive value: 91%
	Roux et al [25], 2008	76 out of 109 positive initial and secondary screenings did have OA 10 out of 140 with a negative initial screening were screened as positive by the rheumatologist; 2 of these were confirmed to have OA	For knee OA: Sensitivity: 87% Specificity: 93% Positive predictive value: 51% Negative predictive value: 98%
	Snoeker et al [26], 2015	Probability of having meniscus tear: With minimum score of 15 points: 8.83% With maximum score of 320 points: 81.5%	Area under the curve: 0.76 (95% CI 0.72-0.80) With a score of 150: Sensitivity: 86.1% Specificity: 45.5% Positive predictive value: 55% Negative predictive value: 81.1% False-negative ratio: 14.1%

^a^OA: osteoarthritis.

### Screening Applications

Ratzlaff et al [24] used a web-based questionnaire to screen for hip and knee OA. The authors used a skip logic method: an affirmative answer to a question would result in a more specific question about that topic, and a negative answer would skip ahead to the next topic. After filling out the questionnaire, all participants were interviewed and examined by an experienced orthopedic physiotherapist in a hospital setting. The physiotherapist used a standardized clinical questionnaire and physical examination to determine the diagnosis. On the basis of a sensitivity of 73% in combination with a specificity of 96%, Ratzlaff et al [24] concluded that these web-based questionnaires can be used to identify hip and knee OA in community- and population-based studies when the purpose is to link potential risk factors to knee and hip health.

Roux et al [25] used a real-life telephone questionnaire with 8 multiple-choice questions that was applied to a random population sample aged between 40 and 75 years to screen for knee and hip OA. A total of 4 questions were aimed at knee OA, and the other 4 questions were aimed at hip OA. The questions focused on the number of days that a patient perceived pain, difficulty in climbing stairs or walking on slopes, walking range, swelling of the knee, and previously diagnosed hip or knee OA. The questionnaire was validated in a previous study [27]. The interview was conducted by a trained interviewer who was a non–health care professional. If the questionnaire was positive, the same telephone questionnaire was repeated by a rheumatologist. The rheumatologist was blinded to the initial outcome of the web-based questionnaire. If the second questionnaire was positive, the diagnosis was confirmed by a physician who knew the patient or the patient was invited for a physical examination and radiographs. A control group with a negative initial questionnaire underwent the same diagnostic procedure. On the basis of 87% sensitivity and 92% specificity, the authors concluded that the telephone questionnaire was able to detect and screen patients with symptomatic OA in the general population [25].

Lim et al [23] developed a screening tool to select patients suspected to have OA. The study used data from a large South Korean database (KNHANES) that was used to monitor the health and nutritional status of approximately 10,000 Koreans since 1998 [23,28]. The investigators selected persons aged >50 years. A DNN was used to look for risk patterns that increased the chance of knee and hip OA and compare this with self-reported previously diagnosed OA. Demographic data, lifestyle, physical activity, and other chronic diseases were used as the input layer for the DNN. On the basis of an AUC of 76.8%, Lim et al [23] concluded that it was possible to detect patients at high risk of OA early in a sample from a large database.

Snoeker et al [26] developed a digital questionnaire screening test for meniscal tears. Possible predictors of meniscal tears were extracted from earlier systematic reviews and used in the initial test. Nonsignificant predictors were deleted via the Least Absolute Shrinkage and Selection Operator procedure, after which the AUC was calculated. The authors used the *best model* from the AUC to develop the screening tool. A false-negative ratio of 15% was accepted as the authors intended to develop a screening tool. The final test contained a questionnaire of 8 items with 7 questions about general demographics (age and gender) as well as more specific questions about the injured knee (weight bearing, effusion, warmth, performance during sport, and discoloration). The last question involved a physical examination to be performed, the deep squat test, under the supervision of a physiotherapist for reliability. Each question could result in a number of points given based on the previously calculated predictors. The higher the number of total points, the higher the probability of having a meniscal tear, with a probability range from 0% to 79%. Snoeker et al [26] concluded based on a false-negative ratio of 14.1% that the test improved the detection of meniscal tears, although further evaluation of the application is needed to assess its usefulness in clinical practice.

### Decision Support Studies

Bisson et al [20] used a web-based questionnaire to establish a differential diagnosis of knee complaints. Questions included age, sex, history of injuries, location of pain, and previous treatments. The program generated secondary questions depending on the answers given to the primary set of questions, a so-called skip logic. The algorithm made a differential diagnosis out of the answered questions with the aim of not missing the correct diagnosis in the differential diagnosis, thus aiming for a high sensitivity. Owing to very similar patient histories, patellar chondromalacia and patellofemoral syndrome, patellar contusion and saphenous nerve contusion, plica syndrome, and trochlear chondromalacia were fused as patellofemoral pain. The same applied to OA and OA exacerbation. After this process, the algorithm could generate a total of 21 possible diagnoses (Textbox 2). The top 3 generated diagnoses were patellofemoral pain, OA, and meniscal tear. Quadriceps tendinitis and stress fracture were the least frequently reported musculoskeletal diseases. Considering its high sensitivity (89%), the authors concluded that the algorithm was an accurate method for generating a differential diagnosis of knee pain [20]. Bisson et al [21] conducted another study using the same algorithm as that in their previous study. In this study, patients had to select the right disorder from the differential diagnosis list generated by the algorithm. The authors added explanatory information to the different diagnoses to aid the patients in selecting the right diagnosis from the list of proposed differential diagnoses. The program generated a mean of 6.6 diagnoses per patient, and patients were able to determine the correct diagnosis from the list generated by the program 58% of the time [21]. The authors concluded that, despite the availability of credible medical resources, there is still no guarantee that the consumer will interpret this information appropriately when forming their own opinion regarding their medical problem, highlighting the importance of a medical provider performing a physical examination as well as any necessary tests.

Elkin et al [22] developed an expert system to establish a differential diagnosis of 12 knee disorders (Textbox 2) to refer patients to the right orthopedic surgeon. The primary data consisted of 26 questions regarding patient history, with 126 possible answers. In total, 2 orthopedic surgeons made a diagnosis based on 469 patient cases. These diagnoses were used as the gold standard in this study. Each of the 126 answers was given a weighting that was used to calculate the probability of having a specific knee disorder. The weighting was calculated using 4 models based on 2 different methods. In model 1, a total of 2 orthopedic surgeons used clinical guidelines, biomedical research, and expert knowledge to assign the weighting to each answer (the so-called Bayesian method). In model 2, the weighting was based solely on the clinical knowledge and experience of the clinician who assigned a weighting to each answer in the question list (the so-called heuristic method). The authors hypothesized that a combination of these 2 methods would generate the most accurate differential diagnosis list. Therefore, model 3 was generated, which included a formula that contained the importance of not missing a disorder (disease importance) and the importance of the answer to the question of having a specific knee disorder (term importance). The values for disease and term importance were provided by orthopedic surgeons based on their clinical experience and knowledge. This algorithm was combined with model 1 to form model 3 and with model 2 to form model 4. Model 3 was the best in including the correct diagnosis within the first 5 diagnoses listed. The expert system, in >95% of the cases, included the true diagnosis in the top 5 diagnoses determined using model 3 but was not able to correctly list the number 1 diagnosis. The authors concluded that, as a correct referral system, model 3 outperformed the other models. However, when using the application as a reminder system, there was no difference between the models as they included the same diagnoses in the top 5.

## Discussion

### Principal Findings

The most important finding of this scoping review is that, although the field of digital health applications is expanding rapidly, the number of peer-reviewed digital applications to establish a remote orthopedic knee diagnosis is limited, with 7 studies included in this review. From the included studies, we were able to provide a descriptive analysis of the currently available applications that are compared with face-to-face contact or conventional imaging. A maximum of 25 knee disorders were included in the studies in this review, of which OA was the most frequent. This is a small number in view of the >1400 knee diagnoses in the International Classification of Diseases, 11th Revision (ICD-11) [29]. None of the studies used wearables as an input parameter for the primary diagnosis. The focus of the studies included in this review was on screening and decision support.

### Evaluation of the Included Studies and Comparison With Other Specialties

In total, 57% (4/7) of the studies focused on screening for a specific knee disorder, of which 75% (3/4) presented a relatively high sensitivity [23-26]. It must be noted that 67% (2/3) of these studies may have been subject to bias because of the following question: “Have you been diagnosed with OA before?” [24,25]. This might have overestimated the sensitivity as there was a high correlation between this question and the correct prediction of knee OA in these studies [24,25]. Thus, the questionnaire may be less sensitive in a general population without previously diagnosed OA. It is interesting that most of the screening studies (4/7, 57%) selected patients from the general population instead of a hospital population. As such, OA screening could be used for early patient education and adequate prevention programs that can delay or avoid referral to a hospital, which in turn might reduce societal costs [30]. These screening tools may not directly assist in establishing a remote diagnosis, but their widespread use may help generate the necessary amount of relevant data to be used as input for future digital health applications. However, at this moment, screening questionnaires and applications are of limited value in establishing a remote diagnosis in clinical practice.

All decision support applications (3/7, 43%) were developed by The State University of New York (Elkin et al [22] and Bisson et al [20,21]). The diagnosis of knee disorders by orthopedic surgeons was reported to be correct in 56% to 80% of the cases [31]. Clinical decision support might be part of the solution to assist in the shortcomings of physicians to establish the primary knee diagnosis [32]. The decision support system by Bisson et al [20,21] was able to include the correct diagnosis in the differential diagnosis list with a sensitivity of 89%. Therefore, it could establish a differential diagnosis to assist the orthopedic surgeon in making a remote diagnosis. The decision support system was not accurate in ranking the different diagnoses.

Clinical decision-making is a complex process that requires information from different sources such as patient history, physical examination, imaging, and laboratory investigations [33]. The studies were accurate in the inclusion of the right diagnosis in the differential diagnosis but not in listing the correct diagnosis first. None of the applications contained information on physical examination, imaging, or laboratory tests. Uploading pictures taken by the patient of swelling or a specific wound has already been integrated in some other medical professions [34]. In addition, imaging or laboratory outcomes obtained from other institutions are currently available for sharing in the digital domain. Decision support accuracy for remote knee diagnosis may improve if these parameters are incorporated into future digital applications.

A potential drawback in the studies by Bisson et al (2/7, 29%) [20,21] was that they included only 21 of the most common knee disorders. Uncommon knee disorders might be missed if the surgeon relies merely on the decision support system [35]. Integrating different types of information may result in better diagnoses and a more reliable ranking of the differential diagnosis. Additional use of validated information from imaging, wearables, gait analysis, smartphones, accelerometers, gyroscopes, and inertial sensors could provide more insights into the dynamic movements of the knee and the influence of complaints in daily living [4,36-41]. Different types of data gathering, such as speech recognition, digital scribes, and serious gaming, could make the patient history more personalized and specific compared with a standard set of questions [42]. AI could be valuable in analyzing these larger data sets and might improve prediction models and provide decision support to a physician [8].

Several AI techniques are being increasingly studied for diagnosis in health care [8,43-45]. In this review, only 29% (2/7) of the studies used AI techniques for knee disorders [22,23]. Elkin et al [22] used AI to establish a differential diagnosis of 10 knee disorders. This was the only study that integrated expert knowledge with statistical input from the literature in the algorithm and compared this with solely expert or statistical knowledge. The model that combined expert knowledge with statistical knowledge (model 3) was the most adequate in showing the correct diagnosis in the top 5 diagnoses. However, it could not rank the diagnoses in the right order. Therefore, this application cannot be used to determine a remote diagnosis but might be able to assist the orthopedic surgeon in making a differential diagnosis. The study by Lim et al [23] was the only study that used deep learning on big data with indirect parameters (eg, lifestyle- and health status–related variables such as smoking status, BMI, alcohol intake, self-reported health status, and medically diagnosed chronic disease) to screen for OA in the general population. Deep learning is a form of machine learning and uses different layers (so-called neurons) to recognize patterns in a data set. A DNN can be trained partially by humans (supervised) or can train itself (unsupervised) to perform different tasks [3,46]. A possible bias in this study was that patients were only included in the model if OA was previously diagnosed.

### Strengths and Limitations

There are some limitations to this scoping review. First, we did not include conference papers, white papers, or abstracts as they were not within the scope of the databases searched. This is because the aim of this review was to focus on the methodology of the available applications and subsequent results, and as such, peer review of the articles was deemed desirable. To decrease the chance of missing relevant articles, the references of the included studies were screened. This did not yield any new articles. Second, a positive publication bias could have led to an underestimation of what has been researched until the present as this study only investigated published research. Lessons learned from developed and tested applications that were not successful in diagnosing knee disorders remotely would have been very valuable in light of the aim of this scoping review. A search of trial registries could have led to a better estimation of positive publication bias. Third, the search strategy excluded gait analyses that were performed in a hospital or research laboratory. It is conceivable that the devices used in these studies could also be applicable outside the hospital and, thus, be valuable for this scoping review. However, we did not find any relevant articles in this category in the references of the included articles. Finally, cultural and geographic factors might limit the applicability of the results. All the included studies (7/7, 100%) were conducted in Europe, North America, and South Korea. As such, they might have limited value in a different global setting such as low- and middle-income countries because of, for example, internet access and cultural differences.

### Future Directions

Several factors could be considered when conducting research in the future regarding digital health applications. To improve future research for the development of digital health applications, there should be a focus on the construction and connection between reliable clinical databases to create big data sets that can be used for machine learning [43,44]. Other medical specialties have already made use of these big data sets and indirect data to detect insomnia for mental disorders and arrhythmias for cardiac diseases [45,47]. Big data sets can be created by integrating different validated digital application modalities such as imaging, questionnaires, wearables, and the use of activity trackers and cameras in mobile phones into 1 model, which may improve the sensitivity and specificity of a diagnosis in orthopedic surgery.

Standardizing the methods and reporting of studies on digital health would be beneficial for future clinical implementation [48]. An essential prerequisite for clinical implementation will be data protection and legal issues regarding privacy-sensitive information transfer and storage by the different applications [49,50]. Encrypted messaging and blockchain may offer opportunities regarding these data issues [50]. Legal responsibilities concerning privacy, liability, and insurance should all be considered when developing a new digital health application [49]. Currently, diagnostic imaging and laboratory studies are almost inconceivable without a digital environment. Progress in data encryption techniques will also likely continue to enhance the protection of the privacy of these data. Therefore, it is logical to assume that future digital health applications will integrate multiple sources of information into 1 application.

This scoping review shows that there are a limited number of available applications to establish a remote diagnosis of knee disorders in orthopedic surgery. To date, there is limited evidence that digital health applications can actually assist a patient or orthopedic surgeon in establishing the primary diagnosis of knee disorders. Future research should aim to integrate multiple sources of information and standardize study designs with close collaboration among clinicians, data scientists, data managers, lawyers, and service users to create reliable and secure databases.

## Appendix

Multimedia Appendix 1 Scoping review checklist page 1.

Multimedia Appendix 2 Scoping review checklist page 2.

Multimedia Appendix 3 Embase and Pubmed search.

Multimedia Appendix 4 Extracted data.

## Abbreviations

AI: artificial intelligence
AUC: area under the curve
DNN: deep neural network
OA: osteoarthritis
PRISMA: Preferred Reporting Items for Systematic Reviews and Meta-Analyses
PRISMA-ScR: Preferred Reporting Items for Systematic Reviews and Meta-Analyses extension for Scoping Reviews

## References

[1] (2019). WHO guideline: recommendations on digital interventions for health system strenghtening. World Health Organization.

[2] (2020). What is Digital Health?. U.S. Food & Drug Administration (FDA).

[3] Ravi D, Wong C, Deligianni F, Berthelot M, Andreu-Perez J, Lo B, Yang G (2017). Deep learning for health informatics. IEEE J Biomed Health Inform.

[4] Agostini M, Moja L, Banzi R, Pistotti V, Tonin P, Venneri A, Turolla A (2015). Telerehabilitation and recovery of motor function: a systematic review and meta-analysis. J Telemed Telecare.

[5] Caldas R, Mundt M, Potthast W, Buarque de Lima Neto F, Markert B (2017). A systematic review of gait analysis methods based on inertial sensors and adaptive algorithms. Gait Posture.

[6] Fu J, Wang Y, Li X, Yu B, Ni M, Chai W, Hao L, Chen J (2018). Robot-assisted vs. conventional unicompartmental knee arthroplasty : systematic review and meta-analysis. Orthopade.

[7] Haeberle HS, Helm JM, Navarro SM, Karnuta JM, Schaffer JL, Callaghan JJ, Mont MA, Kamath AF, Krebs VE, Ramkumar PN (2019). Artificial intelligence and machine learning in lower extremity arthroplasty: a review. J Arthroplasty.

[8] Siebelt M, Das D, Van Den Moosdijk A, Warren T, Van Der Putten P, Van Der Weegen W (2021). Machine learning algorithms trained with pre-hospital acquired history-taking data can accurately differentiate diagnoses in patients with hip complaints. Acta Orthop.

[9] Lawrenz JM, Krout JC, Moran CP, Ready AK, Schafer EA, Higgins RT, Halpern JL, Schwartz HS, Holt GE (2021). Telemedicine in orthopedic oncology during COVID-19: patient satisfaction, reimbursement, and physical examination competency. Orthopedics.

[10] Chaudhry H, Nadeem S, Mundi R (2021). How satisfied are patients and surgeons with telemedicine in orthopaedic care during the COVID-19 pandemic? A systematic review and meta-analysis. Clin Orthop Relat Res.

[11] Iyengar K, Jain VK, Vaishya R (2020). Pitfalls in telemedicine consultations in the era of COVID 19 and how to avoid them. Diabetes Metab Syndr.

[12] Smith E, Hoy DG, Cross M, Vos T, Naghavi M, Buchbinder R, Woolf AD, March L (2014). The global burden of other musculoskeletal disorders: estimates from the Global Burden of Disease 2010 study. Ann Rheum Dis.

[13] Lewis R, Gómez Álvarez CB, Rayman M, Lanham-New S, Woolf A, Mobasheri A (2019). Strategies for optimising musculoskeletal health in the 21st century. BMC Musculoskelet Disord.

[14] (2021). Global strategy on digital health 2020-2025. World Health Organization.

[15] Bunt CW, Jonas CE, Chang JG (2018). Knee pain in adults and adolescents: the initial evaluation. Am Fam Physician.

[16] Turkiewicz A, Gerhardsson de Verdier M, Engström G, Nilsson PM, Mellström C, Lohmander LS, Englund M (2015). Prevalence of knee pain and knee OA in southern Sweden and the proportion that seeks medical care. Rheumatology (Oxford).

[17] GBD 2019 Diseases and Injuries Collaborators (2020). Global burden of 369 diseases and injuries in 204 countries and territories, 1990-2019: a systematic analysis for the Global Burden of Disease Study 2019. Lancet.

[18] Tricco AC, Lillie E, Zarin W, O'Brien KK, Colquhoun H, Levac D, Moher D, Peters MD, Horsley T, Weeks L, Hempel S, Akl EA, Chang C, McGowan J, Stewart L, Hartling L, Aldcroft A, Wilson MG, Garritty C, Lewin S, Godfrey CM, Macdonald MT, Langlois EV, Soares-Weiser K, Moriarty J, Clifford T, Tunçalp Ö, Straus SE (2018). PRISMA extension for scoping reviews (PRISMA-ScR): checklist and explanation. Ann Intern Med.

[19] Ouzzani M, Hammady H, Fedorowicz Z, Elmagarmid A (2016). Rayyan-a web and mobile app for systematic reviews. Syst Rev.

[20] Bisson LJ, Komm JT, Bernas GA, Fineberg MS, Marzo JM, Rauh MA, Smolinski RJ, Wind WM (2014). Accuracy of a computer-based diagnostic program for ambulatory patients with knee pain. Am J Sports Med.

[21] Bisson LJ, Komm JT, Bernas GA, Fineberg MS, Marzo JM, Rauh MA, Smolinski RJ, Wind WM (2016). How accurate are patients at diagnosing the cause of their knee pain with the help of a web-based symptom checker?. Orthop J Sports Med.

[22] Elkin PL, Schlegel DR, Anderson M, Komm J, Ficheur G, Bisson L (2018). Artificial intelligence: Bayesian versus heuristic method for diagnostic decision support. Appl Clin Inform.

[23] Lim J, Kim J, Cheon S (2019). A deep neural network-based method for early detection of osteoarthritis using statistical data. Int J Environ Res Public Health.

[24] Ratzlaff C, Koehoorn M, Cibere J, Kopec J (2012). Clinical validation of an Internet-based questionnaire for ascertaining hip and knee osteoarthritis. Osteoarthritis Cartilage.

[25] Roux CH, Saraux A, Mazieres B, Pouchot J, Morvan J, Fautrel B, Testa J, Fardellone P, Rat AC, Coste J, Guillemin F, Euller-Ziegler L, KHOALA Osteoarthritis Group (2008). Screening for hip and knee osteoarthritis in the general population: predictive value of a questionnaire and prevalence estimates. Ann Rheum Dis.

[26] Snoeker BA, Zwinderman AH, Lucas C, Lindeboom R (2015). A clinical prediction rule for meniscal tears in primary care: development and internal validation using a multicentre study. Br J Gen Pract.

[27] Guillemin F, Saraux A, Fardellone P, Guggenbuhl P, Behier JM, Coste J, Epidemiology Committe of the French Society of Reumatology (2003). Detection of cases of inflammatory rheumatic disorders: performance of a telephone questionnaire designed for use by patient interviewers. Ann Rheum Dis.

[28] Kweon S, Kim Y, Jang MJ, Kim Y, Kim K, Choi S, Chun C, Khang Y, Oh K (2014). Data resource profile: the Korea National Health and Nutrition Examination Survey (KNHANES). Int J Epidemiol.

[29] (2019). ICD-11: International classification of diseases. 11th revision. World Health Organization.

[30] Skou ST, Roos EM (2017). Good Life with osteoArthritis in Denmark (GLA:D™): evidence-based education and supervised neuromuscular exercise delivered by certified physiotherapists nationwide. BMC Musculoskelet Disord.

[31] Nickinson R, Darrah C, Donell S (2010). Accuracy of clinical diagnosis in patients undergoing knee arthroscopy. Int Orthop.

[32] Persson E, Barrafrem K, Meunier A, Tinghög G (2019). The effect of decision fatigue on surgeons' clinical decision making. Health Econ.

[33] Loftus TJ, Tighe PJ, Filiberto AC, Efron PA, Brakenridge SC, Mohr AM, Rashidi P, Upchurch Jr GR, Bihorac A (2020). Artificial intelligence and surgical decision-making. JAMA Surg.

[34] Freeman K, Dinnes J, Chuchu N, Takwoingi Y, Bayliss SE, Matin RN, Jain A, Walter FM, Williams HC, Deeks JJ (2020). Algorithm based smartphone apps to assess risk of skin cancer in adults: systematic review of diagnostic accuracy studies. BMJ.

[35] LaGrandeur K (2020). How safe is our reliance on AI, and should we regulate it?. AI Ethics.

[36] Driscoll M, Fortier-Tougas C, Labelle H, Parent S, Mac-Thiong JM (2014). Evaluation of an apparatus to be combined with a smartphone for the early detection of spinal deformities. Scoliosis.

[37] Fujita K, Watanabe T, Kuroiwa T, Sasaki T, Nimura A, Sugiura Y (2019). A tablet-based app for carpal tunnel syndrome screening: diagnostic case-control study. JMIR Mhealth Uhealth.

[38] Kotti M, Duffell LD, Faisal AA, McGregor AH (2017). Detecting knee osteoarthritis and its discriminating parameters using random forests. Med Eng Phys.

[39] Na A, Buchanan TS (2021). Validating wearable sensors using self-reported instability among patients with knee osteoarthritis. PM R.

[40] Roblot V, Giret Y, Bou Antoun M, Morillot C, Chassin X, Cotten A, Zerbib J, Fournier L (2019). Artificial intelligence to diagnose meniscus tears on MRI. Diagn Interv Imaging.

[41] Rozevink SG, van der Sluis CK, Garzo A, Keller T, Hijmans JM (2021). HoMEcare aRm rehabiLItatioN (MERLIN): telerehabilitation using an unactuated device based on serious games improves the upper limb function in chronic stroke. J Neuroeng Rehabil.

[42] Coiera E, Kocaballi B, Halamka J, Laranjo L (2018). The digital scribe. NPJ Digit Med.

[43] Gulshan V, Peng L, Coram M, Stumpe MC, Wu D, Narayanaswamy A, Venugopalan S, Widner K, Madams T, Cuadros J, Kim R, Raman R, Nelson PC, Mega JL, Webster DR (2016). Development and validation of a deep learning algorithm for detection of diabetic retinopathy in retinal fundus photographs. JAMA.

[44] Handelman GS, Kok HK, Chandra RV, Razavi AH, Lee MJ, Asadi H (2018). eDoctor: machine learning and the future of medicine. J Intern Med.

[45] Perez MV, Mahaffey KW, Hedlin H, Rumsfeld JS, Garcia A, Ferris T, Balasubramanian V, Russo AM, Rajmane A, Cheung L, Hung G, Lee J, Kowey P, Talati N, Nag D, Gummidipundi SE, Beatty A, Hills MT, Desai S, Granger CB, Desai M, Turakhia MP, Apple Heart Study Investigators (2019). Large-scale assessment of a smartwatch to identify atrial fibrillation. N Engl J Med.

[46] Cabitza F, Locoro A, Banfi G (2018). Machine learning in orthopedics: a literature review. Front Bioeng Biotechnol.

[47] Melcher J, Hays R, Torous J (2020). Digital phenotyping for mental health of college students: a clinical review. Evid Based Ment Health.

[48] Aapro M, Bossi P, Dasari A, Fallowfield L, Gascón P, Geller M, Jordan K, Kim J, Martin K, Porzig S (2020). Digital health for optimal supportive care in oncology: benefits, limits, and future perspectives. Support Care Cancer.

[49] Botrugno C (2018). Telemedicine in daily practice: addressing legal challenges while waiting for an EU regulatory framework. Health Policy Technol.

[50] Chenthara S, Ahmed K, Wang H, Whittaker F, Chen Z (2020). Healthchain: a novel framework on privacy preservation of electronic health records using blockchain technology. PLoS One.

