# Supplementary figures and images for: Digital Health Applications to Establish a Remote Diagnosis of Orthopedic Knee Disorders: Scoping Review
Source: J Med Internet Res. 2023 Feb 9;25:e40504. doi: 10.2196/40504 (PMC9951077; doi:10.2196/40504)

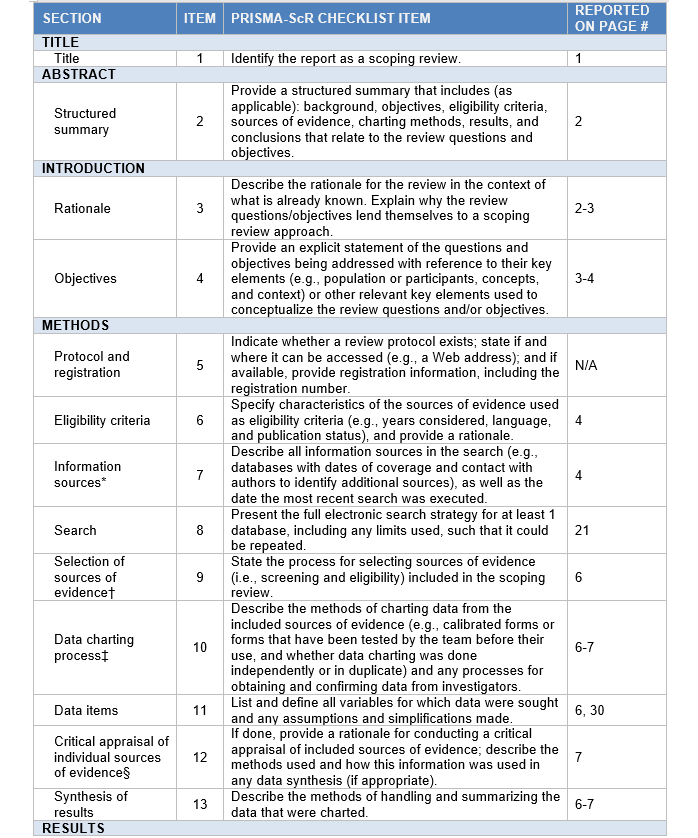

Supplement: Multimedia Appendix 1 [file jmir_v25i1e40504_app1.png]

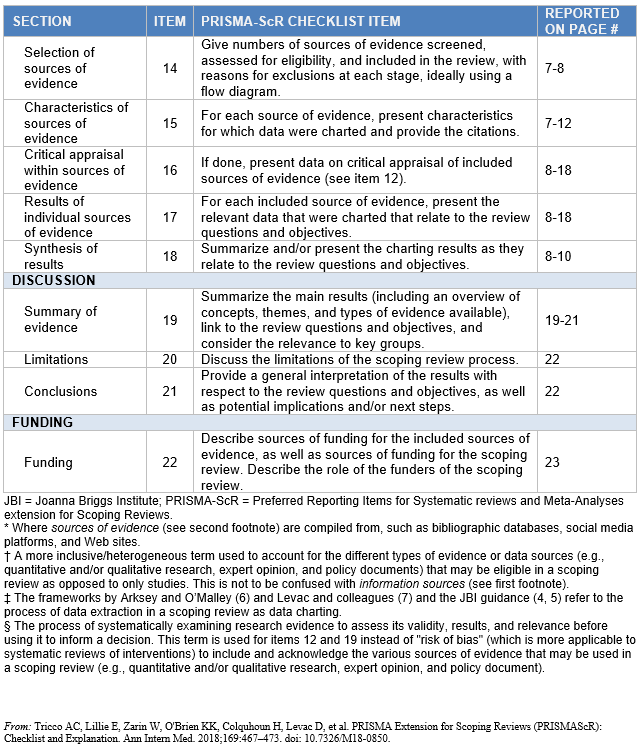

Supplement: Multimedia Appendix 2 [file jmir_v25i1e40504_app2.png]

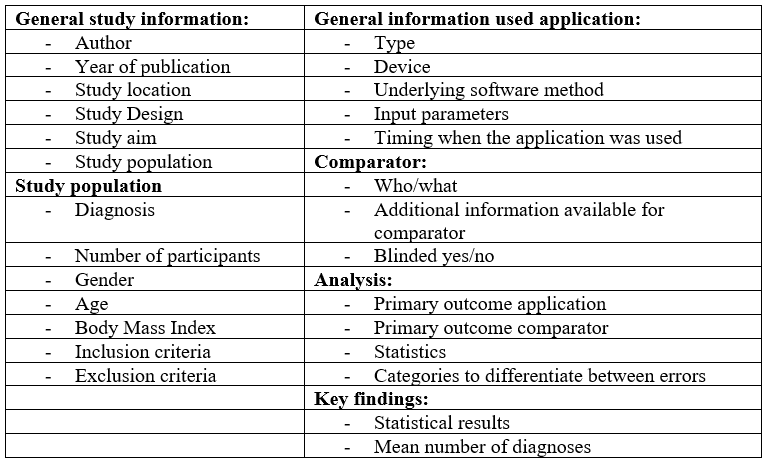

Supplement: Multimedia Appendix 4 [file jmir_v25i1e40504_app4.png]
