# Supplementary material for: Digital Health Applications to Establish a Remote Diagnosis of Orthopedic Knee Disorders: Scoping Review
Source: J Med Internet Res. 2023 Feb 9;25:e40504. doi: 10.2196/40504 (PMC9951077; doi:10.2196/40504)
Supplement: Multimedia Appendix 3 [file jmir_v25i1e40504_app3.docx]

## Appendix 3 Search

**Embase.com, 29-4-2021**

| **No.** | **Query** | **Results** |
| --- | --- | --- |
| #3 | #1 AND #2 | **1598** |
| #2 | 'knee'/exp OR 'articulatic genus':ti,ab,kw OR 'genopathy':ti,ab,kw OR 'knee*':ti,ab,kw OR 'knee compartment*':ti,ab,kw OR 'knee movement*':ti,ab,kw OR 'knee stiffness*':ti,ab,kw OR 'knee pain'/exp OR 'knee pain':ti,ab,kw OR 'painful knee syndrome':ti,ab,kw OR 'knee osteoarthritis'/exp OR 'femorotibial arthrosis':ti,ab,kw OR 'gonarthrosis':ti,ab,kw OR 'knee arthrosis':ti,ab,kw OR 'knee joint arthrosis':ti,ab,kw OR 'knee joint osteoarthritis':ti,ab,kw OR 'knee osteo-arthritis':ti,ab,kw OR 'knee osteo-arthrosis':ti,ab,kw OR 'knee osteoarthritis':ti,ab,kw OR 'knee osteoarthrosis':ti,ab,kw OR 'knee injury'/exp OR 'knee injur*':ti,ab,kw OR 'knee joint injur*':ti,ab,kw OR 'knee joint trauma*':ti,ab,kw OR 'knee open injur*':ti,ab,kw OR 'knee trauma*':ti,ab,kw OR 'knee joint*':ti,ab,kw | **238848** |
| #1 | 'telehealth'/exp OR 'e-health':ti,ab,kw OR 'ehealth':ti,ab,kw OR 'tele-health':ti,ab,kw OR telehealth:ti,ab,kw OR 'telemedicine'/exp OR 'tele medicine':ti,ab,kw OR 'telemedicine':ti,ab,kw OR 'telemonitoring'/exp OR 'remote patient monitoring':ti,ab,kw OR (((tele OR distance OR remote OR patient) NEAR/3 monitoring):ti,ab,kw) OR 'internet'/exp OR 'internet':ti,ab,kw OR 'internet connection*':ti,ab,kw OR 'world wide web':ti,ab,kw OR 'character user interface*':ti,ab,kw OR 'command-line interface*':ti,ab,kw OR 'command-line user interface*':ti,ab,kw OR 'computer interface*':ti,ab,kw OR 'computer user interface*':ti,ab,kw OR 'graphic user interface*':ti,ab,kw OR 'graphical user interface*':ti,ab,kw OR 'text user interface*':ti,ab,kw OR 'text-based user interface*':ti,ab,kw OR 'user computer interface*':ti,ab,kw OR 'user-computer interface*':ti,ab,kw OR 'multimedia'/exp OR 'multimedia':ti,ab,kw OR 'cell phone':ti,ab,kw OR 'cell phones':ti,ab,kw OR 'cellphone':ti,ab,kw OR 'cellphones':ti,ab,kw OR 'cellular phone*':ti,ab,kw OR 'cellular telephone*':ti,ab,kw OR 'telephone screening*':ti,ab,kw OR 'mobile phone*':ti,ab,kw OR 'mobile telephone*':ti,ab,kw OR 'mobile app*':ti,ab,kw OR 'mobile application*':ti,ab,kw OR 'portable software app*':ti,ab,kw OR 'portable software application*':ti,ab,kw OR 'tablet application*':ti,ab,kw OR 'medical informatics'/exp OR 'american recovery and reinvestment act':ti,ab,kw OR 'clinical informatics':ti,ab,kw OR 'clinical information science*':ti,ab,kw OR 'clinical information technolog*':ti,ab,kw OR 'health informatics':ti,ab,kw OR 'health information science*':ti,ab,kw OR 'health information technolog*':ti,ab,kw OR 'medical computer science*':ti,ab,kw OR 'medical data processing':ti,ab,kw OR 'medical informatics':ti,ab,kw OR 'medical informatics applications':ti,ab,kw OR 'medical informatics computing':ti,ab,kw OR 'medical information science':ti,ab,kw OR 'medical information technology':ti,ab,kw OR 'public health informatics':ti,ab,kw OR 'tablet computer'/exp OR 'ipad':ti,ab,kw OR 'laptop/tablet computer':ti,ab,kw OR 'tablet computer*':ti,ab,kw OR 'web-based intervention'/exp OR 'internet-based intervention*':ti,ab,kw OR 'web-based':ti,ab,kw OR 'internet-intervention*':ti,ab,kw OR 'online-based intervention':ti,ab,kw OR 'online-intervention':ti,ab,kw OR 'web intervention*':ti,ab,kw OR 'web-based intervention':ti,ab,kw OR 'deep learning'/exp OR 'deep learning*':ti,ab,kw OR 'hierarchical learning*':ti,ab,kw OR 'whatsapp'/exp OR whatsapp*:ti,ab,kw OR 'wearable sensor'/exp OR 'wearable sensor*':ti,ab,kw OR 'personal digital assistant'/exp OR 'hand held computer*':ti,ab,kw OR 'handheld computer*':ti,ab,kw OR 'palm pc':ti,ab,kw OR 'palmtop':ti,ab,kw OR 'personal data assistant':ti,ab,kw OR 'personal digital assistant':ti,ab,kw OR 'pocket computer*':ti,ab,kw OR 'pocket-sized computer*':ti,ab,kw | **337145** |

Pubmed, 28-4-2021

| **Search** | **Query** | **Results** |
| --- | --- | --- |
| #4 | Search: **18077540 OR 25073597 OR 26962542 OR 29898469 OR 30974803 OR 22975023** Sort by: **Most Recent** | 6 |
| #3 | Search: **#1 AND #2** Sort by: **Most Recent** | 4,041 |
| #2 | Search: **"Knee"[Mesh] OR "Knee Joint"[Mesh] OR "Osteoarthritis, Knee"[Mesh] OR "Knee Injuries"[Mesh] OR "Patellofemoral Pain Syndrome"[Mesh] OR knee*[tiab] OR "superior tibiofibular joint*"[tiab] OR "tibial menisci"[tiab] OR "patellofemoral joint*"[tiab] OR ((patellofemoral[tiab] OR femoropatellar[tiab]) AND (articulation[tiab])) OR (("anterior cruciate"[tiab]) AND (ligament*[tiab])) OR ACL[tiab] OR "Iliotibial band"[tiab] OR "IT band"[tiab] OR "patellar dislocation*"[tiab] OR "genopathy"[tiab] OR "patellofemoral pain*"[tiab] OR "anterior knee*"[tiab] OR "painful knee"[tiab] OR "femorotibial arthrosis"[tiab] OR "gonarthrosis"[tiab] OR ((knee*[tiab]) AND (arthrosis[tiab] OR "joint arthrosis"[tiab] OR osteoarthritis[tiab] OR "osteo-arthrosis"[tiab] OR osteoarthrosis[tiab] OR "joint injur*"[tiab] OR "joint trauma*"[tiab] OR trauma*[tiab] OR injur*[tiab] OR movement*[tiab] OR stiffness* OR pain[tiab] OR joint*[tiab] OR dislocation*[tiab] OR compartment*[tiab]))** | 190,148 |
| #1 | Search: **"Telemedicine"[Mesh] OR "Internet"[Mesh] OR "Multimedia"[Mesh] OR "Medical Informatics"[Mesh] OR "Computers, Handheld"[Mesh] OR "Internet-Based Intervention"[Mesh] OR "tele medicine"[tiab] OR telemedicine[tiab] OR "mobile health"[tiab] OR mhealth[tiab] OR ehealth[tiab] OR "e health"[tiab] OR telemonitoring[tiab] OR "tele monitoring"[tiab] OR internet[tiab] OR "internet connection"[tiab] OR "world wide web"[tiab] OR cyberspace*[tiab] OR "cyber space*"[tiab] OR "tele health"[tiab] OR "telehealth"[tiab] OR "distant monitoring"[tiab] OR "patient monitoring"[tiab] OR ((remote[tiab] OR distan*[tiab]) AND monitoring[tiab]) OR "tele monitoring"[tiab] OR "telemonitoring"[tiab] OR "computer interface*"[tiab] OR "user interface*"[tiab] OR "user interface*"[tiab] OR "multimedia"[tiab] OR "mobile phone*"[tiab] OR "cell phone*"[tiab] OR "cellphone*"[tiab] OR "cellular phone*"[tiab] OR "cellular telephone*"[tiab] OR "mobile phone*"[tiab] OR "mobile telephone*"[tiab] OR "smart phone*"[tiab] OR "mobile application*"[tiab] OR "telephone screening*"[tiab] OR "mobile app*"[tiab] OR "mobile application*"[tiab] OR "portable software"[tiab] OR "tablet application*"[tiab] OR "clinical informatics"[tiab] OR (("clinical information"[tiab]) AND (science*[tiab] OR technolog*[tiab])) OR "health informatics"[tiab] OR (("health information"[tiab]) AND (science*[tiab] OR technolog*[tiab])) OR "medical computer science*"[tiab] OR "medical data"[tiab] OR "medical informatics"[tiab] OR "medical information science"[tiab] OR "medical information technolog*"[tiab] OR "public health informatics"[tiab] OR ipad*[tiab] OR laptop*[tiab] OR "tablet computer*"[tiab] OR "handheld computer*"[tiab] OR "hand held computer*"[tiab] OR "palm top"[tiab] OR palmtop*[tiab] OR "palm PC"[tiab] OR "PDA computer*"[tiab] OR "PC pocket"[tiab] OR "mobile device*"[tiab] OR "palm pilot*"[tiab] OR "internet based intervention"[tiab] OR "internet intervention"[tiab] OR "online based intervention"[tiab] OR "online intervention"[tiab] OR "web intervention"[tiab] OR "web based"[tiab] OR "deep learning"[tiab] OR "hierarchical learning"[tiab] OR whatsapp[tiab] OR "wearable sensor"[tiab] OR "personal data assistant"[tiab] OR "personal digital assistant"[tiab] OR "pocket"[tiab]** Sort by: **Most Recent** | 733,971 |

**Cochrane, 28-4-2021**

| **ID** | **Search** | **Hits** |
| --- | --- | --- |
| **#1** | **knee* OR "knee compartment*" OR "knee movement*" OR "knee stiffness" OR "knee pain" OR "knee osteoarthritis" OR "knee arthrosis" OR "knee joint arthrosis" OR "knee joint osteoarthritis" OR "knee injur*" OR "knee joint*" OR "knee joint trauma*" OR "knee trauma*" OR "gonarthrosis"** | **32614** |
| **#2** | **telehealth OR ehealth OR telemedicine OR telemonitoring OR remote OR "patient monitoring" OR "distance monitoring" OR webbased OR internet** | **31640** |
| **#3** | **#1 AND #2** | **517** |
